# The effect of training intervention based on the theory of planned behavior on oral and dental health behaviors in pregnant women

**DOI:** 10.1186/s12903-023-03239-w

**Published:** 2023-07-25

**Authors:** Fatemeh Mohammadkhah, Razieh Mardani Amirhajelu, Maryam Bakhtiar, Saina Alempour Salemi, Marzieh Kevenjan, Ali Khani Jeihooni

**Affiliations:** 1 Department of Community Health, Child Nursing and Aging, Ramsar School of Nursing, Babol University of Medical Sciences, Babol, Iran; 2 Department of Public Health, School of Health, Fasa University of Medical Sciences, Fasa, Iran; 3 Department of Dental Public Health, School of Dental, Shiraz University of Medical Sciences, Shiraz, Iran; 4 Department of Public Health, School of Health, Shiraz University of Medical Sciences, Shiraz, Iran; 5 Nutrition Research Center, Department of Public Health, School of Health, Shiraz University of Medical Sciences, Shiraz, 7153675541 Iran

**Keywords:** Health training, Theory of planned behavior, Behavior, Pregnant women, Self-care

## Abstract

**Background:**

Pregnancy is a transient physiological condition that causes adverse oral and dental consequences. The present study aimed to determine the effect of a training intervention based on the theory of planned behavior on oral and dental behaviors in pregnant women.

**Methods:**

This quasi-experimental study was conducted on 140 pregnant women (70 in the intervention group and 70 in the control group) supported by comprehensive health centers on the outskirts of Shiraz, Iran, in 2019–2020. The sampling was performed in each center by a simple random method. The tool included a demographic characteristics questionnaire, a questionnaire based on the theory of planned behavior, a self-care behavior questionnaire, and checklists for recording DMFT(Decayed, Missing due to caries, and Filled Teeth(DMFT)) and dental plaque indices. The questionnaires were completed before and 3 months after the intervention by both groups. The intervention group received six 50-min training sessions. The data were analyzed using SPSS 22, the chi-squared test, independent t-test, paired t-test, and descriptive statistics (*p* < 0.05).

**Results:**

The mean ages of the intervention and control groups were 32.28 ± 6.14 and 31.84 ± 6.71, respectively. The results showed that the average scores of all constructs of the theory of planned behavior, dental plaque indices (PI), and MDFT in the intervention group significantly changed after the intervention (*p* < 0.001).

**Conclusion:**

According to the results, training based on the theory of planned behavior was effective on dental and oral health behaviors in pregnant women and improved the clinical results of their self-care behaviors. Therefore, training sessions will increase the knowledge of pregnant women, and providing timely consultations and examinations can be helpful and effective in developing oral and dental health behaviors in pregnant women.

## Background

Oral and dental health are important parts of general health. Neglecting it leads to tooth decay, gingivitis, dental plaque, and periodontitis [1]. Oral and dental health are an important issues for the general health of the pregnant woman and her baby [2]. The relationship between dental diseases and general health is bi-directional in pregnant women and affects their overall condition [3].

The factors that increase the risk of tooth decay during pregnancy include neglecting oral and dental health, consuming sugary things, hormonal changes, changes in oral acidity, nausea, and vomiting. There are hormonal and nutritional changes at the beginning of pregnancy. Oral and dental problems will lead to nutritional disorders, problems in the digestive system and other body systems, premature birth, and low birth weight (LBW) [1]. Periodontitis will lead to LBW/ preterm birth (PTB) new-borns, uterine leiomyoma, gestational hypertension, premature delivery (PTD), and small gestational age [4].

Oral health may be considered an important part of prenatal care. Nearly 60 to 75% of pregnant women have gingivitis. 1 in 4 women of childbearing age have untreated cavities. Children of mothers who have high levels of untreated cavities or tooth loss are more than three times more likely to have cavities as children [5]. Dental caries and gingivitis in pregnant occurred 1.97 times more frequently [6]. The mean DMFT index in women with 1, 2, 3, 4, 5, 6, and more than six pregnancies was obtained at 12.74 ± 7.11, 13.09 ± 7.06, 14.80 ± 7.81, 17.07 ± 8.11, 19.82 ± 9.02, 22.89 ± 8.98, and 26.17 ± 8.01, respectively, and the DMFT index increased by 34% for each unit increase in the number of pregnancies [7]. In Chinese pregnant women, the rates of tooth decay, periodontal health, mass detection, shallow periodontal pocket, deep periodontal pocket, regular oral examination, correct brushing, DMFT index, and caries filling ratio were 69.8%, 1.8%, 95.6%, 51.1%, 4.9%, 22.8%, 49.6%, 2.27%, and 45.4%, respectively. Only 26.3% of them used dental floss once a day, 47.3% washed their mouths more than twice a day, and 46.9% cleaned their tongue every week [8].

In a study in Dubai on 1450 pregnant women, 49.9% of the participants reported at least one dental problem during pregnancy [9]. Gingivitis was also common in Nepalese pregnant women and was associated with the age and height of the mother, the high cost of dental care, and other risk factors among pregnant women in rural areas [10]. Deghatipour et al. conducted a study on 407 pregnant women in the second and third trimesters of pregnancy and showed that their oral and dental health was not satisfactory, and the pregnant women under study on average had 7 decayed teeth in the third trimester of pregnancy [11]. In a study on pregnant women in the city of Arak, Iran, it was indicated that 55.4% of pregnant women had periodontal disease, and brushing frequency was the main predictor of gum health [12]. In another study in the city of Ilam, Iran, it was reported that in total, 30% of pregnant women had healthy gums, and 18% of them had bleeding gums [13].

Pregnancy is a transient physiological condition that brings different hormonal changes to a woman's body and causes adverse oral and dental consequences. Increased knowledge among healthcare professionals and pregnant women will help to avoid and minimize such adverse consequences. Health training is an important means to inform pregnant women of their dental and oral health, contributing to the effective treatment of oral and dental problems during pregnancy [14]. Pregnant women may avoid care concerning oral and dental health due to a lack of knowledge about the importance and effects of oral and dental health during this period, a negative attitude towards oral and dental care during pregnancy, a lack of clinical guidelines for dental management during pregnancy, non-compliance with practical standards, and anxiety about the health of the baby [11]. The results of different studies have indicated that all pregnant women do not have sufficient information about the importance of oral and dental health care during pregnancy, have a low understanding of the benefits of oral and dental health behaviors, are not motivated enough to do such behaviors, and have not received social support [15–17].

Different theories explain human behavior. The theory of planned behavior (TPB), the most valid theory of behavior change, is used to predict, describe, and understand oral and dental health behaviors. According to this theory, all factors that may affect behavior (attitudes, subjective norms, and perceived behavioral control) indirectly affect behavior through behavioral intention [18, 19]. Attitude is defined as a person's feelings about the consequences of health behaviors. Subjective norms are a person's beliefs about a certain behavior that can be influenced by close relationships. Perceived behavioral control refers to a person’s perception of the difficulty of performing a specific oral health behavior. In addition, the constructs of this model are strong predictors of oral health behavioral intention (OHBI) in populations in countries such as Canada, Ireland, Romania, and Iran. Attitudes and perceived behavioral control can also strongly predict oral health goals or behaviors [20].

Considering the importance and status of oral and dental health in pregnant women around the world and in Iran, the role of increased knowledge of pregnant women to avoid and minimize adverse consequences of dental problems, lack of knowledge about the importance and effects of oral and dental health during this period, negative attitude towards oral and dental care during pregnancy, lack of clinical guidelines for dental management during pregnancy, non-compliance with practical standards and anxiety about the health of the baby, low understanding of the benefits of oral and dental health behaviors, low motivated to do dental health behaviors and low social support and the role of training health and TPB in improving knowledge, attitude, motivation, understanding of the benefits of oral and dental health behaviors and improve do dental health behaviors, the present study aimed to determine the effect of training intervention based on the theory of planned behavior on oral and dental health behaviors in pregnant women.

## Methods

The present study was a quasi-experimental study, and the population consisted of pregnant women referring to the comprehensive health centers on the outskirts of Shiraz, Iran, in 2019–2020.

### Sample size and population

According to Khadijah Haji Miri et al.'s study [21], the average difference in dental plaque index reduction between the intervention and comparison groups was 1.21 ± 1.46 and 0.41 ± 0.65, respectively. And considering the power of 90 observations and the first type error of 0.05, the minimum sample size for the study was estimated to be 18 people in each group. In order to increase the power of the study and take into account possible dropouts in each group, 70 people were included in the study.

$${n}_{1}= {n}_{2}= \frac{{\left({{S}_{1}}^{2}+{{S}_{2}}^{2}\right)\left({Z}_{1-\frac{\alpha }{2}}+ {Z}_{1-\beta }\right)}^{2}}{{\left(\overline{{X }_{1}}- \overline{{X }_{2}}\right)}^{2}}$$

Sampling was done in clusters. Out of six treatment centers in Shiraz, two were randomly selected. In each of these two selected centers, 70 people who were available entered the study and were randomly assigned to two intervention and control groups.

**Fig. 1:**
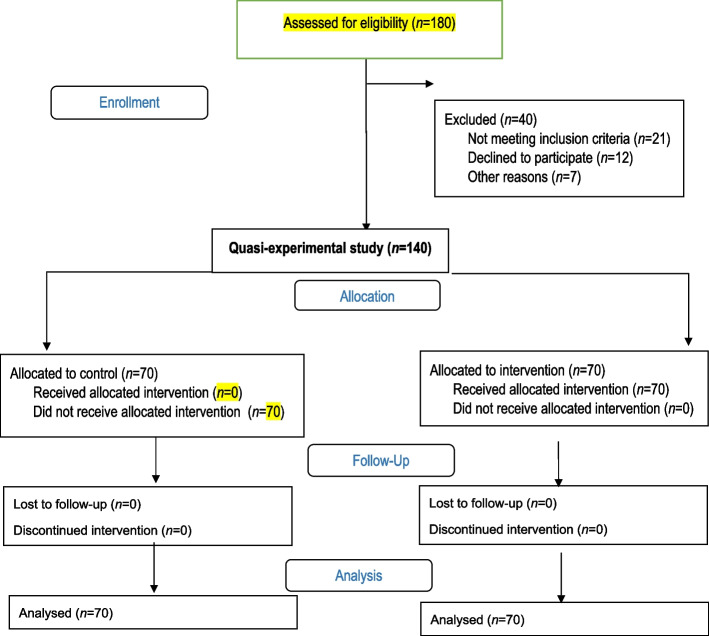
Flow chart of study

Then, the subjects were invited to gather in the center on a special day; they were introduced to each other and explained the goals of the study, and written consent forms were obtained. The inclusion criteria were literacy, the first trimester of gestation, and signing the informed consent form to participate in the study. The exclusion criteria included employment in jobs related to dentistry, suffering from advanced oral and dental diseases, and being absent for more than one training session (Fig. 1).

### Data collection tools

The tools of this research were prepared according to other studies and sources [22–24]. Data collection tools were demographic characteristics (age, duration of pregnancy, number of children, rank of pregnancy, education, monthly income, occupation, living place, spouse’s education), questions related to the constructs of the theory of planned behavior, pregnant women’s performance questionnaires, and checklists for recording DMFT and dental plaque indices.

To evaluate the validity of the questionnaire items, an impact index item higher than 0.15 and a content validity ratio above 0.79 were considered, and based on the exploratory factor analysis, they were classified into six factors. In order to determine the content validity, twelve specialists and professionals (outside the research team) in the fields of health education and health promotion (*n* = 8), gynecology (*n* = 1), dentistry (*n* = 1), community oral health (*n* = 1), and biostatistics (*n* = 1) were consulted. Then, according to the Lawshe table, items with a content validity ratio (CVR) higher than 0.56 were considered acceptable and retained for the subsequent analysis [25]. To assess the reliability of the instrument, a cross-sectional study was conducted on 200 pregnant women over the age of 20 in healthcare centers in Shiraz city. Then, the reliability of the instrument was assessed using the internal consistency method. The overall Cronbach’s alpha was 0.89. Moreover, the Cronbach’s alpha was 0.87 for awareness, 0.84 for attitude, 0.89 for subjective norms, 0.90 for perceived behavioral control, 0.88 for behavioral intention, and 0.89 for oral and dental health behaviors.

Ten items were asked about awareness (3-option items that were scored between 0 and 2), eight items were about attitude (for example, using dental floss is effective in preventing the formation of plaque) (the items were scored between 8 and 40), eight items were about perceived behavioral control (for example, I’m sure that to have healthy teeth every night before bed) (the items were scored between 8 and 40), and eight questions were asked about behavioral intention (for example, I intend to floss my teeth at least once a day) (they were scored between 8 and 40). The attitude items were scored between 1 and 5 using a 5-point Likert scale. The performance checklist also included 12 items (in the form of yes or no items) on different areas such as the way to brush teeth, flossing, visiting the dentist regularly, and using fluoride mouthwash after nausea and vomiting. The first items were measured and recorded through direct observation of the mother's performance on the mouth-teeth model, and the other items were self-reported. The minimum score was 0, and the maximum score was 12. The checklists for recording DMFT and dental plaque were recorded by the dentist at the health center.

### Procedure

Before the intervention, the data were collected from both the intervention and control groups, and then the training intervention was performed for the intervention group. The women in the intervention group received routine pregnancy care and participated in the training sessions, while the women in the control group received only routine pregnancy care.

Six 50-min training sessions were held for the intervention group in the form of group discussion, question–answer, using pictures and pamphlets, practical presentations using mouth and teeth models, toothbrushes, dental floss, and playing films and PowerPoint presentations. The training program was implemented by a Ph.D. in health education and promotion and two dentists in collaboration with women's healthcare experts and an oral hygienist. The sessions were held for seven groups of ten (70 people in the intervention group) once a week. The contents included information about the structure of the mouth and teeth and the surrounding tissues; information about the characteristics of healthy and diseased gums; the symptoms and complications of oral and dental diseases during pregnancy; increasing the skills of the correct brushing and flossing techniques; and providing all women under study with a toothbrush, toothpaste, and dental floss. To increase the mother's ability, successful models, i.e., other women who could keep their teeth healthy due to observing oral and dental health care, as well as using training videos, the mothers’ performance skills in the correct techniques of brushing and flossing were improved through using practical presentations mouth-teeth models and training videos, pamphlets, and posters. It should be noted that at the end of each session, the presented materials were provided to the participants in the form of pamphlets and training booklets for study, and before starting each session, the materials presented in the previous session were briefly reviewed. In this study, the dental plaque was determined by the dentist using Navy Plaque Index (NPI). The validity of this index has been confirmed by researchers in previous studies [26]. The diagnosis of tooth decay was done by the tactile-visual method, i.e., the methods proposed by the WHO, with a dental mirror, sickle probe and natural light.

Data collection methods included interviews and examinations. The plaque index (PI) was measured by a close-up index. The DMFT checklist was also filled out by the dentist through examination and the number of decayed teeth (DT), missed teeth (MT), and filled teeth (FT). In this study, the DMFT index included a) decayed: when there is a discoloration on the smooth surface of the teeth, or there is a lesion inside the pits, fissures, and grooves that is emptied under the enamel, or the surrounding area of the lesion is soft. A tooth was considered decayed because not only did it have visible signs of enamel decay, but it was also completely soft in contact with the tip of the probe, and the probe got stuck in it during the examination. If a tooth was covered with plaque that prevented the detection of decay, it was first cleaned with sterile gas and then examined for the state of decay; b) missed tooth: the tooth that was missed due to decay; c) filled tooth: the teeth that were filled or repaired due to decay [27]. The dental plaque index was also examined by the dentist and measured in percentage. One session was held in the presence of women's spouses and the authorities of the healthcare centers; they were provided with oral and dental health behaviors, and their support roles and effective subjective norms were emphasized. At the end of the session, an educational booklet and CD were given to the pregnant women and their spouses. The participants in the intervention group received training and a motivational short text message once a week to maintain and improve their activities. A virtual group was created on WhatsApp to communicate information. Two virtual follow-up sessions were held one and two months after the intervention. Three months after the intervention, the data for both groups were collected. At the end of the study, the control group was given an educational booklet.

### Ethical considerations

To comply with ethical considerations, permission was obtained from the Ethics Committee of Fasa University of Medical Sciences [IR.FUMS.REC.1395.76] and healthcare centers on the outskirts of Shiraz, and the women under study have explained the goals and necessity of the project and assured that the information would be confidential. The data were analyzed using SPSS 22, the chi-square test, independent t-test, and paired t-test, and the significance level was considered to be 0.05.

## Results

One Hundred Forty pregnant women (70 in the intervention group and 70 in the control group) participated in this study. The mean age of the women in the intervention group and control group was 32.28 ± 6.14 and 31.84 ± 6.71, respectively, which was not significantly different between the two groups according to the results of the independent t-test (*p* = 0.326).

The average duration of pregnancy in the intervention and control groups was 9 ± 3 and 10 ± 2, respectively, which was not significantly different between the two groups according to the results of the independent t-test (*p* = 0.304).

The results of the chi-square test showed no significant difference between the two groups in terms of the variables of the number of children, rank of pregnancy, education, monthly income, occupation, living place, and spouse's education (Table 1).

**Table 1:** Comparison of demographic variables of pregnant women under study in the two intervention and control groups

Variable		Intervention group		Control group		*P*-value
		No	Percentage	No	Percentage	
Occupation	Housewife	61	87.14	65	92.86	0.259
	Employed	9	12.86	5	7.14	
Family monthly income	Under 50 million rials	33	47.14	36	51.43	0.184
	50–100 million rials	30	42.86	29	41.43	
	Above 100 million rials	7	10	5	7.14	
Number of children	0	15	21.43	13	18.57	0.159
	1	34	48.57	31	44.29	
	2	18	25.71	22	31.43	
	3	2	2.86	4	5.71	
	4	1	1.43	0	0	
Rank of pregnancy	1	15	21.43	13	18.57	0.165
	2	41	58.57	40	57.14	
	3	12	17.14	14	20	
	4	2	2.86	3	4.29	
Education	Elementary school	8	11.43	6	8.57	
	Secondary school	24	34.28	22	31.43	
	High school	29	41.43	37	52.86	
	Academic	9	12.86	5	7.14	
Living place	Rental	30	42.86	36	51.43	0.155
	Private	40	57.14	34	48.57	
Spouse’s education	Elementary school	5	7.14	8	11.43	0.144
	Secondary school	20	28.57	14	20	
	High school	35	50	40	57.14	
	Academic	10	14.29	8	11.43	

The results showed that there was no significant difference between the two groups in terms of awareness, attitude, subjective norms, perceived behavioral control, performance of oral and dental health behaviors, and dental plaque index (PI), but three months after the intervention, the intervention group showed a significant increase in the above-mentioned variables as compared to the control group (Table 2).

**Table 2:** Comparison of mean scores of awareness, attitude, subjective norms, perceived behavioral control, behavioral intention, the performance of oral and dental health behaviors, and dental plaque index of pregnant women in the two intervention and control groups before and three months after the intervention

Variable	Group	Before the intervention M ± SD	Three months after the intervention M ± SD	*P*-value
Awareness	Intervention	8.45 ± 1.28	16.80 ± 1.36	0.001
	Control	9.80 ± 1.17	10.04 ± 1.20	0.289
	*P*-value	0.236	0.001	
Attitude	Intervention	18.28 ± 3.35	34.70 ± 3.38	0.001
	Control	19.62 ± 3.19	21.35 ± 3.22	0.201
	*P*-value	0.228	0.001	
Subjective norms	Intervention	16.52 ± 3.48	28.20 ± 3.10	0.001
	Control	18.58 ± 3.47	18.79 ± 3.50	0.292
	*P*-value	0.188	0.001	
Perceived behavioral control	Intervention	18.12 ± 3.58	35.80 ± 3.71	0.001
	Control	17.23 ± 3.54	18.44 ± 3.60	0.240
	*P*-value	0.245	0.001	
Behavioral intention	Intervention	19.48 ± 3.88	36.16 ± 3.08	0.001
	Control	20.60 ± 3.78	21.79 ± 3.80	0.249
	*P*-value	0.251	0.001	
Performance of oral and dental health care behaviors	Intervention	5.08 ± 0.26	10.32 ± 0.68	0.001
	Control	5.16 ± 0.29	5.22 ± 0.32	0.212
	*P*-value	0.272	0.001	
Dental plaque index (PI)	Intervention	65 ± 12	34 ± 77	0.001
	Control	63 ± 13	67 ± 11	0.135
	*P*-value	0.273	0.001	
DMFT index	Intervention	5.8 ± 2.2	5.3 ± 1.8	0.228
	Control	5.4 ± 2.3	5.5 ± 2.1	0.269
	*P*-value	0.242	0.321	

## Discussion

The present study aimed to determine the effect of a training intervention based on the theory of planned behavior on oral and dental health behaviors in pregnant women. The results of the study indicated the effectiveness of training based on TPB in improving oral and dental health in pregnant women. This result was in line with the results of the studies by Ebrahimipour et al. (2016), Karimy et al. (2020), and Lee et al. (2020) [22, 28, 29]. In the present study, the intervention group received six training sessions in the form of group discussion, question–answer, educational pamphlets and pictures, a practical presentation using a mouth-teeth model, a toothbrush, and toothpaste, and educational films and PowerPoint presentations, leading to the improvement of oral and dental health behaviors in pregnant women.

The results of this study indicated the effect of training on improving the construct of awareness in the intervention group, which was in line with the results of studies by Abdulelah Almoayad et al. (2021), Mokarrami et al. (2019), and Ahmadi et al. (2020) [30–32].

The results of the study showed that before the intervention, there was no significant difference in the construct of attitude between the two groups, but after the intervention, this construct significantly increased in the intervention group. Group discussion and talking about negative and positive experiences in this regard motivated the participants to model oral and dental health behaviors. To improve the participants' attitudes, an educational book was given to them, which, along with an educational pamphlet and pictures, could improve the attitude in the intervention group. This result was in line with the results of studies by Farzaneh et al. (2021), Naseri-Salahshour et al. (2019), and Pauley et al. (2018) [23, 33, 34]. After the intervention, the mean score of subjective norms significantly increased in the intervention group. In the present study, in a session with the presence of women's husbands and the authorities of the healthcare centers, the husbands learned about oral and dental health behaviors, and their support role and the effective abstract norm were emphasized, which was effective in improving the construct of subjective norms. This result was consistent with the studies of Shojaei et al. (2022), Khani-Jeihooni et al. (2020), and Archila-Godinez et al. (2022) [35–37].

The results of the study showed that before the intervention, there was no significant difference between the two groups in the construct of perceived behavioral control, but after the intervention, it significantly increased in the intervention group. In the present study, using successful examples, i.e., mothers who had healthy teeth due to adherence to oral and dental health care, and educational films led to increased abilities among the participants. Regarding training practical skills, the participants' practical skills in the correct techniques of using dental floss and toothbrush increased using training films, pictures, pamphlets, and posters. It should be noted that at the end of each session, the materials were given to the participants in the form of educational pamphlets and booklets, and before each session, the contents of the previous session were briefly reviewed, which led to an increase in the construct of perceived behavioral control in the intervention group. This result was in line with the results of studies by Armoon et al. (2021), Shaikh-Ahmadi et al. (2019), and Ashtarian et al. (2018) [38–40].

According to the results, there was a significant statistical change in the construct of behavioral intention after the intervention, indicating the effectiveness of the training program in improving behavioral intention in the intervention group as well as increasing other constructs. The constructs of this theory were the most important predictors of behavioral intention [24, 41]. Therefore, it seemed that the effect of a training intervention on improving other constructs of the theory consequently improved the construct of behavioral intention. This result was in line with the studies of Peyman et al. (2015), Amin et al. (2014), and Sqeeney et al. (2015) [42–44].

After the intervention, the pregnant women’s performance in oral and dental health behaviors significantly increased. This result was in line with the results of studies by Lin et al. (2019), Farzaneh et al. (2021), and Ebrahimipour et al. (2016) [22, 23, 45]. It seems that the present study improved pregnant women's oral and dental health behaviors by increasing their knowledge about the importance of oral and dental health as well as their attitudes, perceived behavioral control, and subjective norms. As indicated in previous similar studies, other constructs of TPB are the most important predictors of people’s performance [46–48]. In addition, in the present study, the participants in the intervention group received an educational and motivational short text message once a week to improve their activities, and two follow-up sessions were virtually held for the participants one and two months after the intervention, which seemed to have great importance in improving women’s oral and dental health behaviors.

In this study, the dental plaque was determined with the Navy Plaque Index (NPI) by the dentist before and after the intervention, indicating a significant decrease in the dental plaque index (PI) in the intervention group, which was in line with the results of studies by Simpariano et al. (2017), Lee et al. (2020), and Al Khamis et al. (2017) [29, 49, 50]. One of the limitations of the present study was that the patients’ behaviors could not be observed and the data were self-reported. This problem was solved to some extent by examining the clinical results of the intervention, i.e., the PI and DMFT indexes. A strength of the present study was its theory-based and problem-based interventions for oral and dental health behaviors. Another strength was examining the clinical results of the intervention (PI and DMFT index) in the intervention group.

## Conclusion

According to the results of the present study, it can be concluded that TPB-based health training had a significant effect on increasing the mean scores of all constructs of the theory and improving pregnant women's performance in oral and dental health behaviors and PI. Therefore, it is suggested that healthcare authorities take steps to create and promote self-care behaviors in this target group by considering relevant theory-based training programs, using related educational materials for pregnant women, and following the executive guidelines and protocols of healthcare centers. Politicians and officials can play an effective role in this serious matter by establishing supportive health policies, providing preventive and therapeutic equipment for oral and dental health in healthcare centers, and making amendments to dental insurance tariffs in the public and private sectors.

## Data Availability

The datasets used and/or analysed during the current study available from the corresponding author on reasonable request.

## Abbreviations

OHBI: Oral health behavioral intentions
TPB: Theory of planned behavior
PI: Dental plaque indices
DMFT: Decayed, Missing, Filled, Teeth
